# Yeast Probiotic and Yeast Products in Enhancing Livestock Feeds Utilization and Performance: An Overview

**DOI:** 10.3390/jof8111191

**Published:** 2022-11-11

**Authors:** Yuanxiang Pang, Hailiang Zhang, Haoyu Wen, Hongbing Wan, Hao Wu, Ying Chen, Shengshuo Li, Le Zhang, Xiaojie Sun, Bichen Li, Xuelian Liu

**Affiliations:** 1 State Key Laboratory of Direct-Fed Microbial Engineering, Beijing 100192, China; 2 School of Biological Sciences, The University of Manchester, Manchester M13 9PL, UK

**Keywords:** yeast specie, yeast products, livestock and poultry, ruminant

## Abstract

The intensive use of antibiotics as growth-promoting agents in animal production has resulted in the spread of animal antibiotic resistance and possibly human antibiotic resistance. Based on this premise, it is significant to explore an alternative approach to preventing infectious diseases and promoting animal growth and health. Yeast as the main natural growth promoter in livestock nutrition has been extensively studied for decades. Numerous yeasts and yeast-containing products are produced, marketed, and used in animal feed as providers of nutrient sources, probiotics, and nutrients or serve distinct nutritional functions. A large amount of scientific research suggests that yeasts and their derivatives may be good for animal growth performance and health, especially when animals are housed in poor sanitation or are suffering from disease. However, when yeasts are used as a surrogate for livestock antibiotics, the results vary according to several factors, including yeast species, yeast product components, feed ingredients, animal category, type of symptoms, and differences in the rearing environment. In this review, the effects of different yeasts on different animals will be reviewed. The types of widely used yeast products, their functional characteristics, and application effects will be discussed in order to provide a reference for the development and application of yeast feed products.

## 1. Introduction

The intensive use of antibiotics as growth-promoting agents in animal production has resulted in the spread of animal antibiotic resistance and possibly human antibiotic resistance. Because the use of antibiotics poses a biological threat to human and animal health, the European Union proposed banning the use of antibiotics as a growth promoter and prophylactic measure in 2006 [1]. In areas where antibiotics have been banned, diminished growth performance and increased mortality of animals have been reported as a result of diseases such as salmonellosis and candidiasis [2,3]. Based on this premise, it is significant to explore an alternative approach to preventing infectious diseases and promoting animal growth and health. Growth-promoting agents such as probiotics can alter the intestinal microbial environment, thereby affecting lipid metabolism and, in turn, animal quality. Probiotics can modulate the microbiota of the rumen or hindgut, alter the fermentation pattern, and increase the flow of nutrients to the small intestine, thereby improving the digestible nature of the diet [4]. Some researchers believe that adding probiotics to livestock feed reduced cholesterol levels by bacterial direct uptake of cholesterol compounds [5]. Thus, probiotic supplementation can boost the digestion of fibrin by improving nutritional intake, raising livestock health care, and increasing livestock product yields [6].

One such probiotic that has generated considerable interest in research is yeast. Yeast as the main natural growth promoter in livestock nutrition has been extensively studied for decades. The beneficial effects of yeast in animals are largely due to the fact that some nutrients from yeast products can be used by rumen microbes to increase the number of microorganisms and other effects on the overall health and performance of the animal [7]. Several modes of action have shown that yeast has the potential to absorb O_2_ from the rumen, making the ecological environment more conducive to the growth of rumen anaerobes. It is particularly beneficial in high-fiber diets by increasing rumen cellular decomposition and nutrient digestion. Yeasts regulate pH and reduce acidosis by regulating lactate production. Yeasts contain abundant peptides, vitamins, organic acids, and cofactors that are used by bacteria [6,8,9]. However, when yeasts are used as a surrogate for livestock antibiotics, the results vary according to several factors, including yeast species, yeast product components, feed ingredients, animal category, type of symptoms, and differences in the rearing environment.

In this review, the effects of different yeasts on different animals will be reviewed. The types of widely used yeast products, their functional characteristics, and application effects will be discussed in order to provide a reference for the development and application of yeast feed products.

## 2. Yeast Species

Yeasts are abundant and almost everywhere in the environment. They are isolated in fruit, honey, soil, water, and plant stems, leaves, and flowers [10], and naturally found in the composition of feed, such as cereals, food co-products, pollen, and pollen used to feed animals. Yeasts have about 100 different genera and consist of about 2000 different species, with different cellular morphology, metabolism of different substrates, and different reproductive processes [11]. Most yeast species have no advantages or disadvantages for humans and animals, while some species provide beneficial effects, such as *Saccharomyces cerevisiae*, *Kluyveromyces marxianus*, *Candida utilis* and *Saccharomyces boulardii* [12,13,14,15].

### 2.1. Saccharomyces cerevisiae

Among all the yeast species, *S. cerevisiae* has been used the most in animal production and nutrition [16,17]. *S. cerevisiae* is rich in proteins, polysaccharides, amino acids, small peptides, nucleotides, vitamins, trace elements, and some unknown growth factors. It has many functions, such as improving production performance, promoting intestinal development, regulating intestinal flora balance, improving immunity, and improving meat quality [18]. It is widely used in pig, poultry, and ruminant production.

#### 2.1.1. Probiotic Yeast for Pigs

Although early weaning can bring higher economic benefits, it can also cause a stress response in piglets, so that the intestinal flora of piglets becomes unbalanced, resulting in stunted growth. *S. cerevisiae* is a beneficial fungus, which can inhibit the proliferation of pathogenic bacteria through effective competition and maintain the balance of intestinal flora so that piglets can obtain more nutrients. Wang et al. showed that compared with the control group, diets supplemented with 300 mL/kg *S. cerevisiae* fermentation broth significantly increased average daily gain, and average daily feed intake, while the ratio of feed to gain was significantly decreased. In addition, the contents of total protein, DNA, and RNA of the duodenum, jejunum, and ileum mucosa were significantly increased, villus height/crypt depth of duodenum, jejunum, and ileum as well as IgA, IgG, and IgM of mucosa were significantly increased, which indicated that *S. cerevisiae* can improve the performance of weaned piglets, promote the development of small intestine and enhance mucosal immune function, thus relieving weaning stress of piglets [18,19].

#### 2.1.2. Probiotic Yeast for Poultry

The microbial communities in the chickens’ digestive tract are critical for intestinal homeostasis, and host metabolism, and affect the physiological function and health of the chickens. They play significant roles in digestive nutrition, inhibition of pathogens, interaction with each other, and interaction with the intestine-associated immune system [20]. The major defense against enteric pathogens in the digestive tract is the intestinal microbiota. Disorders in the interaction of the gut microbiota with the host play an important role in the pathogenesis of intestinal disease. There were significant changes in the cecal flora of chickens infected with *C. perfringens* [21,22,23], *Eimeria* species, and *Salmonella Enteritidis* [24,25,26,27,28,29]. In poultry, the benefits of yeast probiotic supplements include increasing the production performance of broilers and increasing the resistance of chickens to enteric pathogens (*Salmonella*, *Campylobacter jejuni*, *C. perfringens*, or *E. coli*) [30,31,32,33]. In addition, yeast probiotic supplementation significantly reduced the colonization rate of *Salmonellae* [34]. More important, *S. cerevisiae* cultures under different fermentation conditions had different effects on the growth performance and immune function of broilers [35].

Yeast dietary supplements for birds and ducks can modulate the intestinal microbiota, inhibit bacterial colonization of the gastrointestinal tract, enhance immune responses, and improve the characterization of animal meat (Table 1).

#### 2.1.3. Probiotic Yeast for Ruminants

Adding *S. cerevisiae* to beef cattle and dairy cows can not only improve performance and immune function, but also reduce nitrogen emission and improve milk quality. Gao et al. showed that adding 0.50% *S. cerevisiae* in Simmental crossbred beef cattle diet can increase the daily gain by 9.68%, decrease feed/gain by 1.79% and increase the economic benefit by 9.09% [47]. Chen et al. reported that dietary supplementation of 240 g/kg *S. cerevisiae* significantly reduced crude protein excretion and plasma total cholesterol concentration of Xiangzhong black cattle, thus reducing nitrogen emission in the beef cattle breeding process [48]. Adding 2% *S. cerevisiae* to the concentrate of Holstein lactating dairy cows significantly increased serum immunoglobulin content, while the serum amino acid transferase activity was significantly reduced [49].

In addition, *S. cerevisiae* can improve the microbial environment, enhance rumen fermentation function, stabilize rumen pH value, promote lactic acid bacteria metabolism, enhance the activity of fibrinolytic enzymes in the rumen, and promote the degradation of fiber substances [50]. Tian et al. reported that stimulation of 200 μg/mL *S. cerevisiae* cell wall significantly increased the mRNA level and protein expression of SDR-1 [51], which indicated that *S. cerevisiae* cell wall could regulate the expression of defensin in epithelial cells and inhibit the proliferation of harmful bacteria, thus regulating rumen function.

### 2.2. Kluyveromyces marxianus

*Kluyveromyces marxianus* is a hemiascomycetous non-conventional yeast, widely existing in the edible natural environment. *K. marxianus* has the advantages of high-temperature resistance, fast growth rate, and wide substrate spectrum compared to traditional yeasts [52]. Moreover, *K. marxianus* has a strong enzyme production capacity, such as inulinase, lipase, galactosidase, glucosidase, carboxypeptidase, aminopeptidase, and lactase. However, there are few reports on the application of *K. marxianus* in livestock and poultry breeding. Zhou et al. showed that the addition of *K. marxianus* significantly improved the growth performance, blood biochemical index, and antioxidant capacity and improved the intestinal structure of broiler chickens [53]. Malinee et al. reported that concentrations of AFM1 in milk were reduced with *K. marxianus*. In addition, supplementation of *K. marxianus* also improved dry matter intake (DMI) and milk compositions, which indicated *K. marxianus* showed promise as a feed additive to detoxify AFB1 and improve DMI and yield of milk components [54].

### 2.3. Candida utilis

*Candida utilis* has been reclassified as *Cyberlindnera jadiniiis*. It is a yeast that has been shown to be a cytoprotein-rich microorganism that improves the balance of intestinal microorganisms and facilitates host growth [55]. *C. utilis* can produce edible protein for humans and livestock by using waste sulfite liquid, wood hydrolysate, and molasses from the paper industry. At present, *C. utilis* is widely used in the aquaculture industry, while there are few reports in livestock and poultry. *C. utilis* not only improved growth capacity and reduced diarrhea in weaning piglets, but also increased the diversity and abundance of cecum microspores in weaning piglets [56]. Jalasutram et al. reported that 48% of the protein content was derived from *C. utilis* digested and undigested poultry [57]. Similar reports have been reported in cattle [55].

### 2.4. Saccharomyces boulardii

*Saccharomyces boulardii* is a subspecies of *S. cerevisiae*, with high-temperature resistance, acid resistance, bile salt resistance, and antioxidant characteristics [58]. Compared with *S. cerevisiae*, *S. boulardii* showed a higher survival rate, higher tolerance to bile salts, and better antioxidant properties under different temperatures and gastric acid conditions [59]. *S. boulardii* is widely used in animals, water production, and other fields because of its good effects in preventing diarrhea of young animals, improving body immunity, improving intestinal barrier function, and improving the performance of livestock and poultry.

#### 2.4.1. Probiotic Yeast for Pigs

*S. boulardii* can effectively reduce the colonization and translocation of pathogenic bacteria in the intestinal tract of pigs, increase IgA antibody secretion in the intestinal tract of pigs, inhibit inflammation, reduce diarrhea and mortality, increase food intake, reduce the feed-to-meat ratio, increase growth performance and improve meat quality, etc. The digestibility, absorption, and immune function of weaned piglets were weak. The average daily gain of piglets in the *S. boulardii* group was 39.9% higher than that in the control group, and the mortality of piglets induced by *E. coli* lipopolysaccharide was reduced by 20% [60]. *S. boulardii* also can regulate the number of lymphocytes, increase IgA antibody secretion in the intestinal tract, and reduce ETEC localization to the intestinal membrane system [61]. In addition, *S. boulardii* increased the number of white blood cells, lymphocytes, and neutrophils in serum and the mass concentration of IFN-γ, and decreased the contents of IL-1 β and IL-6, thereby effectively reducing the diarrhea rate and mortality rate of piglets (20%) [62]. At the same time, *S. boulardii* significantly increased the feeding rate, intestinal organic acid content, intestinal digestibility, feed-to-meat ratio, and growth energy of pigs [63].

#### 2.4.2. Probiotic Yeast for Poultry

The role of *S. boulardii* in poultry is mainly reflected in enhancing the immune recognition and immune regulation function of poultry, reducing the colonization of intestinal pathogens and the number of pathogenic bacteria in feces, increasing the intestinal enzyme content, promoting digestion and absorption of poultry, enhancing the antioxidant capacity of the body, reducing the ratio of feed to meat, so as to increase the quality of broilers. Broilers fed the diet containing 100 million CFU/kg *S. boulardii* significantly increased body weight, the relative weight of the bursa of Fabricius and thymus gland, and the number of immunoglobulin A (IgA) positive cells in the jejunum [64]. In addition, the activities of Na+-K + ATPase, lipase, and γ-glutamyl transpeptidase in the jejunum and the activities of glutathione peroxidase, glutathione reductase, and digestive enzyme in the serum of broilers were significantly increased, while the contents of malondialdehyde, uric acid and triacylglycerol were decreased [65]. Reports showed that *S. boulardii* also significantly increased the villus height, width, goblet cell number, and intestinal ultrastructure of broilers [66].

#### 2.4.3. Probiotic Yeast for Ruminants

The effects of *S. boulardii* on ruminants mainly include stabilizing the rumen environment, improving digestive enzyme activity, improving immunity and antioxidant capacity, and reducing the diarrhea rate. Adding 1 g/d (2.0 × 10^10^ CFU/g) *S. boulardii* to the diet of lactating dairy cows effectively improved feed intake and milk yield [67]. Similarly, sheep fed with *S. boulardii* had higher rumen fibrinolytic enzyme activity, decreased rumen ammonia nitrogen concentration, and increased volatile fatty acid concentration [68]. The report showed that the oxidative burst and phagocytic activity of neutrophil and serum amyloid A2 and C-reactive protein concentrations were increased before and after weaning after feeding calves with *S. boulardii* [69]. *S. boulardii* also enhanced the antibody response in stressed cattle and the immune response to the vaccine in sheep [70,71]. In addition, the supplementation of 1 × 10^10^ CFU/d *S. boulardii* reduced the diarrhea rate from 69.1% to 50.0% and alleviated diarrhea effectively (28.65% vs. 9.5%), which was better than antibiotics [72].

## 3. Yeast Products

Yeast and yeast by-products can be used in livestock production systems. There are many types of yeast supplements, such as active yeast, yeast cell wall (YCW), purified cell wall components, and yeast cultures or extracts after yeast fermentation. These yeast supplements are different in appearance, the composition of biologically active components, and their application to the production system. Additionally, yeast breeding conditions or fermentation conditions, as well as yeast serotypes or strains, have a significant influence on the outcomes of the final product and the after-feeding of livestock. Therefore, it is essential to understand the differences in alternative products in the selection of yeast supplements.

### 3.1. Viable Yeast

Active yeast products are routinely added to animal feed to demonstrate their potential probiotic effects. The most common viable yeast used in the animal feed industry is active dry yeast, which contains approximately 95% of dry matter [13]. As a feed additive for livestock and poultry breeding, active dry yeast has higher microbial activity, which enables it to play a better role in regulating intestinal flora, inhibiting the propagation of harmful bacteria, improving the activity of intracellular enzymes, and promoting the digestion and absorption of nutrients by livestock and poultry. Dietary supplementation with heat-resistant active yeast number (>10^10^ CFU) significantly improved the feed intake and performance of weaned piglets. In addition, the ratio of CD4+/CD8+ in blood and T-lymphocyte conversion rate also significantly increased, which indicated that active dry yeast can effectively inhibit diarrhea of weaned piglets and improve the immunity of weaned piglets [73]. Guo showed that after feeding active dry yeast, the probiotics such as *bifidobacteria* in the stomach of sows significantly increased, which effectively improved the intestinal health of sows [74]. However, all feed must be pasteurized after African swine fever (ASF). High-temperature disinfection seriously reduces the activity of active dry yeast and other microbial products, affecting their effects [75,76]. Meanwhile, due to the lack of activity detection technology for active yeast products sold on the market, it is impossible to evaluate their activity grade, resulting in uneven product effects.

### 3.2. Yeast Cell Wall

The yeast cell walls are mainly composed of glucan (35~45%), mannan oligosaccharides (40~45%), protein (5~10%), chitin (1~2%), lipid (3~8%), and inorganic salt (1~3%). Speranda et al. reported that feeding piglets with *S. cerevisiae* cell wall can promote the proliferation of lymphocytes such as neutrophils and improve their immunity [77]. Polysaccharides contained in yeast cell walls have many biological functions such as enhancing immunity, improving antigenicity of pathogenic substances, relieving stress, and promoting growth and development. At the same time, yeast products have the characteristics of no residue, no drug resistance, and no pollution to the environment. Therefore, yeast cell walls can be used as a green additive in feed, and the main components of this product are mannan oligosaccharide and β-glucan [78].

#### 3.2.1. Mannan Oligosaccharide

Mannan oligosaccharide (MOS) can promote the development of the animal gastrointestinal tract, regulate intestinal flora, improve animal immunity, and improve animal growth performance [79,80,81]. In addition, MOS is resistant to high temperatures and keeps its structure and function intact under high-temperature and high-pressure feed processing and pelleting conditions, which is also conducive to its application as a feed additive in livestock and poultry breeding [82]. Xie et al. found that adding MOS to feed significantly increased the contents of IgM, LBP, IL-6, and SAA in sheep serum, effectively enhancing immune capacity and alleviating inflammation [79]. Yan et al. showed that the T cell conversion rate, macrophage phagocytosis index, and serum Newcastle disease antibody titer of broilers were significantly increased after supplementation of 0.1% *S. cerevisiae* MOS, which suggested that MOS effectively enhanced body immunity [80]. In addition, adding MOS to the diet increased the feed conversion rate of weaned piglets and reduced the diarrhea rate caused by *E.coli* [81].

#### 3.2.2. β-Glucan

β-glucan has the biological functions of promoting the development of gastrointestinal mucosa, balancing intestinal flora, promoting the development of immune organs, enhancing body immunity, promoting feeding, and improving production performance [83,84,85]. Zhou et al. reported that the addition of 75 mg/kg yeast β-glucan increased the height of small intestinal villus, regulated the structure of intestinal flora, and improved the immune function of early weaned calves [83]. Li showed that adding 0.05% β-glucan significantly increased the feed intake and average daily gain of meat rabbits, and at the same time increased the contents of IgG, IgA, IgM antibodies, and immune organ index in serum, so as to improve the body immunity [84]. In addition, similar results have been reported in mice [85,86,87].

### 3.3. Yeast Culture

Yeast cultures are unique among yeast products because they contain yeast organisms and fermented metabolites that are produced during specific fermentation processes. Yeast cultures provide nutrition, enhance intestinal digestive enzyme activity, promote digestion and absorption, and enhance metabolic activities through their metabolites [88,89,90]. Gao et al. pointed out that adding appropriate yeast culture to the diet significantly improved the digestibility of calcium and phosphorus for broilers. More important, yeast culture increased the humoral and cellular immunity of broilers and alleviated the inhibitory effect of coccidiosis on the performance of broilers [88]. The stress ability of weaning piglets was enhanced after yeast culture supplementation, while the number of panting or dying piglets was decreased [89]. The activities of xylanase, endoglucanase, fibrinoglycogenase, and cellulase in the rumen of beef cattle were significantly increased by adding yeast culture to the feeding diet, which indicated that yeast culture improved the digestibility of ruminants by increasing the enzyme activity in the rumen [90]. However, due to the complex composition of yeast culture, different sources of strain, uncertain mechanism of action, and different intestinal physiological environments, the effect of yeast culture produced by the same strain on different animals is different.

### 3.4. Other Yeast Products

In addition to the above main yeast products, some specific yeast products are also being used in a variety of feed industries, including selenium yeast, chromium yeast, and *Phaffia* yeast. At present, there are many reports on selenium-rich yeast. Selenium yeast, as a highly bioavailable selenium sulfate product and market, has a unique role in regulating animal metabolism, improving animal health, and increasing serine content in meat and eggs compared with inorganic feeding [91,92,93,94]. Chromium yeast contains trivalent chromium in combination with biologically active peptides, amino acids, and niacin, which seems to function as a “glucose tolerance factor” acting as a physiological potentiator of insulin action to improve carbohydrate metabolism [95]. Studies of chromium yeast have limited information on animal growth capacity, reproductive biology mechanisms, and effects. Dan et al. showed that the use of chromium yeast to replenish bovine diets reduced morbidity and improved growth after heat stress [96]. *Phaffia* yeast produces a red pigment or carotenoid (astaxanthin). Although the source of astaxanthin is more expensive than the synthesized carotenoids, their bioavailability may be higher because it is related to the organic matrix [97].

## 4. Conclusions

Numerous yeasts and yeast-containing products are produced, marketed, and used in animal feed as providers of nutrient sources, probiotics, and nutrients or serve distinct nutritional functions. A large amount of scientific research suggests that yeasts and their derivatives may be good for animal growth performance and health, especially when animals are housed in poor sanitation or are suffering from disease. However, there are still limitations on yeast products. First, the industry production standards and product testing technology are not standardized. Different enterprises have different production processes, leading to uneven product quality, and different batches of products have different nutritional values. A second area is that there is no specific strain for one animal, and the same strain works differently on different animals. Finally, there is a lack of information regarding the application of yeast products at different stages of production. Further research is needed to better understand yeast and its derivatives in livestock.

## Figures and Tables

**Table 1:** Effect of yeast supplementation in poultry diets with *Saccharomyces cerevisiae*.

*S. cerevisiae* Doses	Poultry Species	Impacts	References
0.1, 0.2, and 0.3% in powdered form	Broiler	Minor serum cholesterol levels, higher serum high-density lipoprotein concentrations	Gheisari [36]
1 g/kg of feed	Quail	Lesser deleterious effects of aflatoxins	Parlat [37]
2 kg/ton of feed	Broiler	Higher numbers of ileal LAB, lesser counts of ileal E. coli	Koc [38]
1, 2 and 3 g/kg of feed	Broiler	Lesser final body weight and daily weight gain, higher feed conversion	Ahmed [39]
0.5 g/kg of feed	Broiler	Lesser serum concentration of nitric oxide, lesser serum myeloperoxidase activity	Wang [40]
0.5 g/kg of feed	Broiler	Minor serum diamine oxidase activity, higher ratio of villus height to crypt depth in the ileum	Wang [41]
200, 250 and 300 mg/kg of feed	Broiler	Lesser feed conversion ratio, lesser mortality	Oyedeji [42]
3 kg/ton of feed	Broiler	Lower blood cholesterol concentrations	Abdelrahman [43]
5 × 10^9^ cells/L drinking water	Broiler	Higher total serum protein levels, serum albumin levels	Pizzolitto [44]
2.5% and 5.0% novel *S. cerevisiae* culture	White Pekin ducks	Higher feed/gain ratio, serum IgM and IgA	Shimin [45]
2.5% novel *S. cerevisiae* culture	White Pekin ducks	Improve meat quality	Shimin [46]
